# Adaptive filtering method for reducing signal saturation induced artifacts in X-ray dark-field tomography

**DOI:** 10.1038/s41598-026-56857-z

**Published:** 2026-06-10

**Authors:** Henrik Mäkinen, Heikki Suhonen, Simo Huotari

**Affiliations:** Department of Physics, University of Helsinki, P.O. Box 64, FI-00014 Helsinki, Finland

**Keywords:** X-ray imaging, Tomography, Talbot-Lau, Dark-field, Phase-contrast, Engineering, Optics and photonics, Physics

## Abstract

X-ray dark-field imaging provides information of an object based on its small-angle scattering properties. Dark-field contrast originates from differences in the strength of scattering from micron and sub-micron sized structures in the object. A Talbot-Lau interferometer can be utilized for dark-field imaging with regular X-ray tubes. A known difficulty with the method arises, when the scattering is too strong, preventing the dark-field and phase retrieval to work accurately. This is similar to a strongly absorbing object in attenuation-based X-ray imaging, and causes problems in tomographic reconstruction that depends on the accuracy of the projected signal. In this article, we present an adaptive filtering approach for the dark-field and phase-contrast projections to improve the signal-to-noise ratio in image areas where the dark-field signal is close to saturation. This filtering improves the projection images markedly, and enables tomographic reconstruction even in cases where the sample contains strong scatterers.

## Introduction

X-ray dark-field imaging exploits the (ultra-) small-angle scattering properties of an object. This scattering arises from structures sized below the resolution of conventional imaging systems – in the micrometer and sub-micrometer scale. These kinds of structures usually remain unresolved with regular X-ray imaging based on attenuation or refraction^1^, which is why the dark-field contrast provides complementary information of the sample compared to those methods. Dark-field imaging can be used to visualize granular structures and fibers, and gain insight on the porosity of the material. Importantly, the dark-field signal can be evaluated quantitatively^2–4^. X-ray dark-field imaging has several applications in both nondestructive testing^5–8^ and biomedical investigations (see, e.g.,^9,10^). The most promising clinically relevant applications are in lung imaging^11,12^ and mammography^13^, with potential for clinical dark-field computed tomography^14^ (CT). Moreover, the orientational dependence of the dark-field signal^15^ makes it suitable for tensor tomography^16,17^.

In the last decades, several methods for X-ray dark-field imaging were developed, including grating interferometry^18^, edge illumination or coded aperture based imaging^19^, and speckle-based imaging^20^. Several variations of grating interferometers exist from more conventional three-grating Talbot-Lau interferometers^1,18^ (TLI) to systems utilizing fewer gratings, tailored for tunable dark-field contrast^21,22^.

**Fig. 1:**
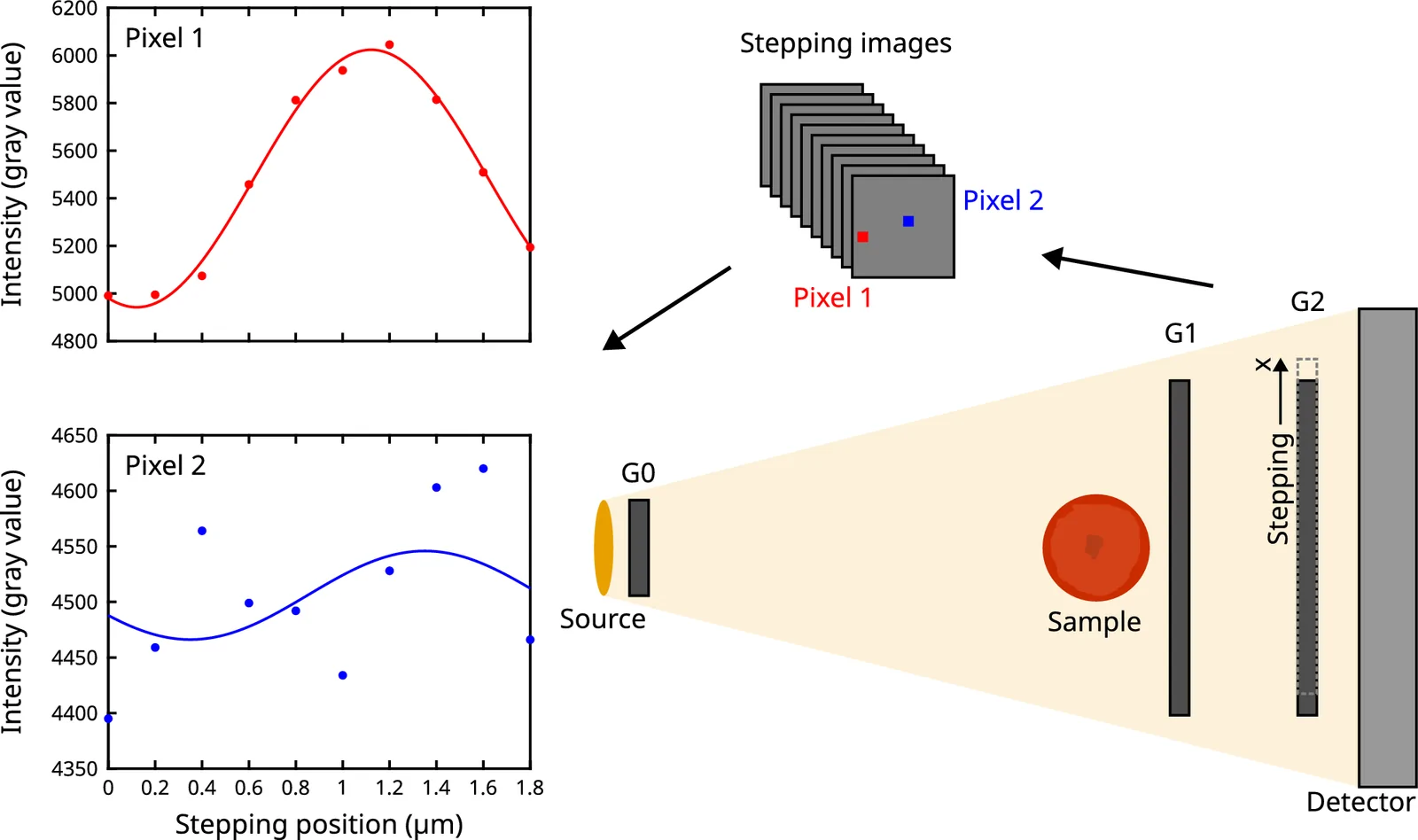
Talbot-Lau interferometer system and phase stepping procedure. The system consists of an X-ray tube source, a detector, the sample, and three diffraction gratings (G0, G1, G2). In a phase stepping scan, the G2 grating is stepped over at least one full grating period in equidistant steps, while an image is acquired at each step. As a result, each image pixel location in the stepping series constitutes a, usually periodic, phase stepping signal. When the phase stepping signal is from an image region with high visibility (Pixel 1), a sinusoidal function is reliably fitted to the sampled stepping points. When the signal originates from a pixel location with reduced visibility due to strong scattering (Pixel 2), a sinusoidal function is not properly defined by the data. In this case, neither the dark-field signal (amplitude of the curve) nor the refraction signal (phase of the curve) can be extracted accurately. Note the different y-axis scales of the plots.

A Talbot-Lau interferometer, also utilized in this work, can be used to simultaneously acquire attenuation, phase-shift and dark-field images, even with regular low-brilliance X-ray tubes. For larger (mm-sized) X-ray source spots, an absorption grating (G0) is utilized to translate the extended source to an array of narrow and individually coherent line sources that satisfy the spatial coherence requirements of the interferometer. A phase-shifting diffraction grating (G1) is then used to create periodic interference patterns at distinct distances downstream, following from the fractional Talbot effect^23,24^. The interference oscillations, with a period of a few micrometers, are sampled with an additional absorption grating (G2) and a retrieval method, such as *phase stepping*^25^ (see Fig. 1). In this process, the interference fringes are essentially translated to approximately sinusoidal intensity modulations in each image pixel location (*m*, *n*):

1 $$\begin{aligned} I(m,n,\Delta x_i) = I_0 T(m,n) \{1 + V(m,n) \sin [\phi (m,n)+2\pi \Delta x_i / p_2]\} + \epsilon (m,n), \end{aligned}$$

where $I_0$ is the incoming intensity (photons/pixel/exposure), *T* is the transmission through the sample, *V* is the *visibility*^18^ of the oscillating signal, *ϕ* is the angular shift of the outgoing beam, $p_2$ is the period of the analyzer grating in the same units as the grating displacement $\Delta x_i$, and *ε* is additive random noise.

Transmission, differential phase and dark-field images can be extracted by comparing the amplitude and phase coefficients of the *stepping curves* (defined by equation (1)) obtained with (*s*) and without (*r*) the sample:

2 $$\begin{aligned} T(m,n)&= \frac{T_s(m,n)}{T_r(m,n)} \end{aligned}$$

3 $$\begin{aligned} \Delta \phi (m,n)&= \phi _s(m,n)-\phi _r(m,n) \end{aligned}$$

4 $$\begin{aligned} D(m,n)&= \frac{V_s(m,n)}{V_r(m,n)}. \end{aligned}$$

The visibility of the interference pattern decreases exponentially with sample thickness^2,26^, in a similar way as the transmission behaves in regular X-ray imaging. A three-dimensional map of the scattering strength can thus be reconstructed from the dark-field projections with regular tomographic reconstruction algorithms, such as filtered back projection.

While the dark-field image retrieval with a TLI is a relatively robust process, several factors, including vibrational instabilities^27^, imperfect phase stepping^28^, and beam hardening^29^, can cause imperfections to the images. Another problem, the one that we will investigate in this paper, originates from strongly scattering regions in the sample, which can result in the saturation of the dark-field signal. Saturation occurs when the statistical noise becomes higher than the contrast/visibility of the measured interference fringes. Figure 1 visualizes the sampled interference fringes (i.e., the phase stepping curves) of a weakly (Pixel 1) and a strongly (Pixel 2) scattering region of an object. In the case of weak scattering, the intensity modulation has a large amplitude and the sinusoidal shape is well defined. In the presence of strong scattering, however, the amplitude is much lower and the sinusoidal shape is practically indistinguishable, and likely misinterpreted. Thus, areas of strong scattering appear as random noise in the dark-field projections, exhibiting a low signal-to-noise ratio (SNR). As the phase-contrast signal based on the phase shift of the stepping curve relies on the visibility of the interference fringes, saturation of the dark-field signal also negatively affects the phase-contrast images. Reconstructed slices from tomographic measurements exhibit severe streaking artifacts, hiding surrounding features of interest.

This issue is analogous to *photon starvation* in regular attenuation-based X-ray imaging^30,31^, where strongly attenuating object regions (such as metal implants) result in insufficient photons reaching the image detector. The noise in these areas is amplified in the tomographic reconstruction process, leading to streak artifacts. Several methods for avoiding photon starvation effects have been presented, including automatic tube current modulation^32,33^ (clinical systems), iterative reconstruction algorithms^34–36^, and pre-processing methods, such as local filtering of the raw data^31,37,38^.

Also the saturation problem arising from strongly scattering sample regions has been identified before^9,26,39,40^. In general, the strength of the dark-field signal is determined by the X-ray energy, the so called autocorrelation length of the system, and the size of the structures in the sample^26^. The autocorrelation length depends on the X-ray wavelength *λ*, the distance between the sample and analyzer grating *L*, and the period of the analyzer grating $p_2$:

5 $$\begin{aligned} \xi = \frac{\lambda L}{p_2}. \end{aligned}$$

By increasing the X-ray energy or varying the sample–detector distance, the measured strength of the scattering can be reduced to avoid signal saturation. Depending on the sample (feature sizes) and imaging system, these adjustments might not be sufficient to fully eliminate the issue, and can lead to other undesired effects. For example, increasing the X-ray energy results in decreased contrast also in the attenuation and refraction images. In asymmetric Talbot-Lau setups, where the distance between the interferometer gratings is short, only a limited range for tuning the autocorrelation length is available. Moreover, adjusting the position of the sample in an otherwise fixed system, will also affect phase-contrast sensitivity^41^ and geometric magnification.

**Fig. 2:**
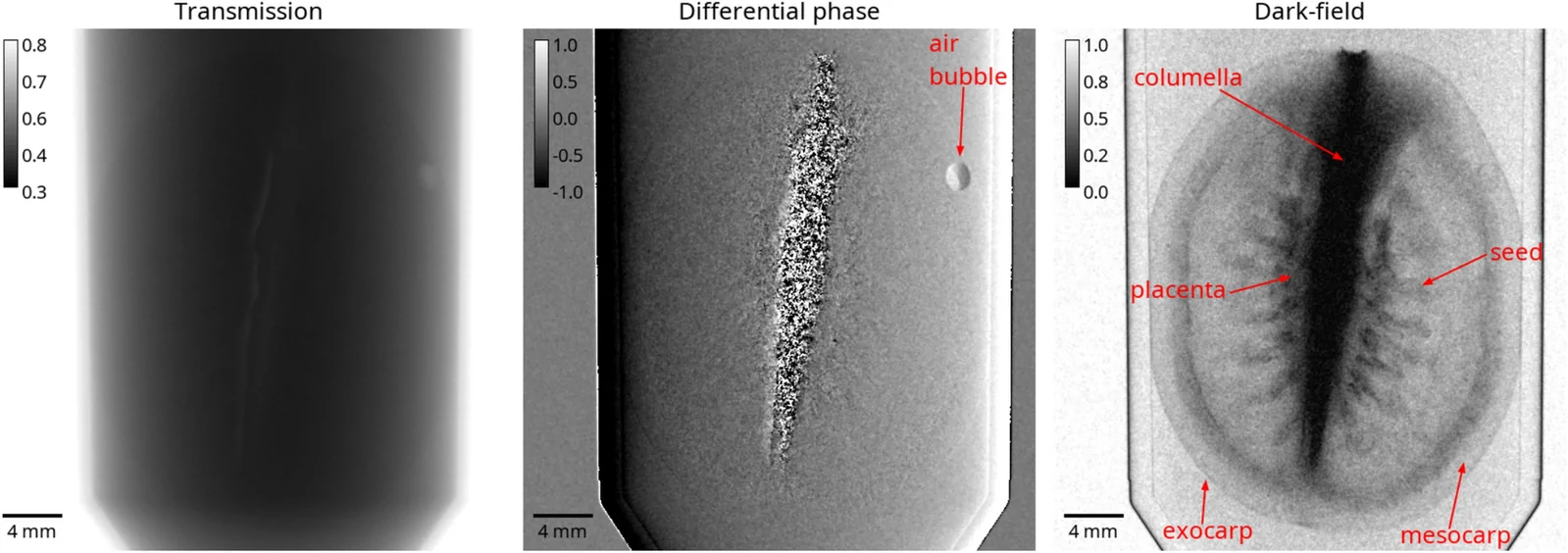
Transmission, differential phase-contrast, and dark-field projections of a mini-plum tomato in a plastic tube filled with water. While in the transmission and phase-contrast images the contrast is low, the dark-field projection manages to visualize all the main structures of the tomato: the outer skin (exocarp), inner skin (mesocarp), the placental tissue, the seeds, and the columnar central part (columella). The dark saturated signal in the dark-field projection is seen as noise in the differential phase projection.

In this work we develop an image processing method for reducing the artifacts in dark-field and phase-contrast images arising from strongly scattering objects. We do this by adaptively filtering the raw image projections before retrieving the visibility and phase. The local filtering strength in the image is controlled by the local SNR value of the dark-field signal. An adjustable parameter, $\textrm{SNR}_{D_0}$, is defined for controlling the general strength of the filter. We demonstrate the method’s applicability by using a mini-plum tomato as the sample. Neither attenuation nor refraction projections can properly visualize the inner structures of the tomato, as presented in Fig. 2. Only from the dark-field signal, all main structures can be identified. The columnar middle part of the tomato (columella), however, strongly scatters X-rays and saturates the dark-field signal. The proposed method significantly reduces streaking artifacts in the reconstructed dark-field and phase-contrast data, with the trade-off of locally reduced spatial resolution.

## Methods

### Adaptive filtering

The purpose of the filtering is to increase the SNR of the dark-field and differential phase-contrast projections in areas where it is low. This filtering will be done on the raw phase stepping images, by doing a weighted average of the stepping curves in neighboring pixels. As a downside, this filtering results in a loss of spatial resolution, essentially blurring the images. The adaptive nature of the filter is aimed at keeping the spatial resolution at the best possible level, so that filtering strength can be chosen locally to match the required SNR level. We will first define the SNR for the dark-field image ($\textrm{SNR}_D$), which we will use as a criterion for determining the required level of filtering, then the effect of the filter on $\textrm{SNR}_D$, and finally we will describe the adaptive approach using an image pyramid.

The parameters *T*, *ϕ*, and *V* in equation (1) are calculated from the intensity signal by fitting a sinusoidal function to the measured data points, usually by doing a Fourier analysis on the data^25^. More advanced extraction methods (e.g.,^4,42^) are needed with irregularly spaced phase step locations. In general, the quality of the fit depends on how much signal there is compared to noise.

We concentrate our analysis on the ideal case where the photon counting statistics are the dominant source of the noise and where the phase step locations are regularly spaced. In this case the Fourier analysis method can be used, and the noise level in each pixel is determined by the number of photons arriving at that pixel. As photons arriving to a pixel are independent events, the photons follow Poisson distribution statistics. At high average photon numbers this can be well approximated by the Gaussian distribution with the standard deviation of the value being equal to the square root of the average number of the arriving photons, i.e., *ε* is drawn from a Gaussian distribution with mean 0, and standard deviation of $\sqrt{I(m,n)}$. For lower photon numbers, the full treatment with Poisson statistics would be more appropriate. We make here the assumption that the average transmission is still relatively good, and the inability to get a reliable fit for equation (1) arises from the strong scattering leading to reduction in the visibility *V*, so that the Gaussian approximation for the counting statistics is reasonably valid.

As a figure of merit, we use the $\mathrm{SNR}$ of the dark-field signal with respect to the noise from the detector counting statistics as defined by Revol et al.^43^ We employ the simplification that the detector noise response is 1, both with and without sample, meaning that the intensities behave as true counts of incoming photons, which would be the case for an ideal detector. Later in the processing we will introduce a free scaling parameter, which controls the overall strength of the filtering, and replaces the true detector response that would otherwise have to be characterized separately for each experimental setup. With this, we arrive at the formulation (pixel indices *m*, *n* dropped for clarity)

6 $$\begin{aligned} \textrm{SNR}_D = \frac{D}{\Delta D} = \frac{V_r\sqrt{N_\textrm{ps}I_0T}}{\sqrt{V_r^2 \left( T+1\right) + 2\left( T+\frac{1}{D^2}\right) }}. \end{aligned}$$

Experimentally, $\textrm{SNR}_D$ could be increased by either increasing the number of phase steps $N_\textrm{ps}$, or increasing the number of photons $I_0$. Tuning the setup to increase the visibility values $V_r$ and $V_s$, or the transmission *T*, could also lead to an increase in the SNR.

In an image processing approach, the $\textrm{SNR}_D$ values could be increased by filtering the dark-field images, typically at the expense of spatial resolution. The term $\sqrt{I_0T}$ in equation (6) originates from the counting statistics of the individual stepping images, and this could be improved by considering combined signals from neighboring pixels in each image of the stepping series. We will focus on improving this term, and thus consider the filtering of the raw stepping images instead of the extracted projections directly.

Digital image filtering for image *f*(*m*, *n*) employing a weighted average of the neighboring pixels can be implemented using a convolution operation, giving the following expression for the filtered image *g*(*m*, *n*):

7 $$\begin{aligned} g(m,n) = h(m,n) \circledast f(m,n). \end{aligned}$$

Here *h*(*m*, *n*) is the convolution kernel, and *⊛* depicts the two-dimensional discrete convolution operation. The effect of such a filter on the $\mathrm{SNR}$ of the image can be calculated using error propagation for a weighted sum, and gives the result

8 $$\begin{aligned} \textrm{SNR}_{g}(m,n) = \frac{g(m,n)}{\Delta g (m,n)} = \frac{\sum _k \sum _l h(k,l) f(m-k,n-l)}{\sqrt{\sum _k \sum _l h(k,l)^2 f(m-k,n-l)}}. \end{aligned}$$

This has used the approximation that $\Delta f = \sqrt{f}$, which is valid for sufficiently high intensities in our idealized detector model. When further assuming that *f* is constant within the support of the convolution kernel, equation (8) can be approximated as

9 $$\begin{aligned} \textrm{SNR}_{g}(m,n) \approx \sqrt{f(m,n)} \frac{\sum _k \sum _l h(k,l)}{\sqrt{\sum _k \sum _l h(k,l)^2 }} = \textrm{SNR}_f(m,n) \frac{\sum _k \sum _l h(k,l)}{\sqrt{\sum _k \sum _l h(k,l)^2 }}. \end{aligned}$$

Here $\textrm{SNR}_f = f/\Delta f = f/\sqrt{f} = \sqrt{f}$ is the signal-to-noise ratio in the unfiltered raw image.

Out of many possibilities for smoothing filters, we will employ a Gaussian kernel with standard deviation $\sigma _{\textrm{filter}}$ to produce the filtered intensity images. This choice is based on the fact that a Gaussian filter is very well studied, and it has a relatively simple analytical form. The discrete Gaussian convolution kernel can be written as

10 $$\begin{aligned} h(m,n) = C\exp \left[ -\frac{m^2+n^2}{2\sigma _{\textrm{filter}}^2} \right] , \end{aligned}$$

where *C* is chosen so that the sum of the kernel values is 1. The increase in $\mathrm{SNR}$ can then be calculated using equation (9). Replacing the discrete kernel with continuous Gaussian distributions to simplify the calculation, we arrive at

11 $$\begin{aligned} \textrm{SNR}_{g}(m,n) = \sigma _{\textrm{filter}} \sqrt{2\pi } \cdot \textrm{SNR}_f(m,n). \end{aligned}$$

The trade-off of such filtering is that it will blur the image even in the areas where an $\mathrm{SNR}$ improvement would not have been needed. If the blur in the original image is of Gaussian form, with $\sigma _\textrm{orig}$, then the filtered image will have a blur of $\sigma = \sqrt{\sigma _\textrm{orig}^2+\sigma _\textrm{filter}^2}$. To solve this issue, we will choose $\sigma _{\textrm{filter}}$ individually for each pixel so that a preset minimum $\textrm{SNR}_D$ value, $\textrm{SNR}_{D_0}$, is achieved at every location of the dark-field image. Starting from equation (6) and inserting the definition for the SNR of the filtered intensity image (equation (11)), we find that at each pixel location

12 $$\begin{aligned} \sigma _{\textrm{filter}}(m,n) = \frac{1}{\sqrt{2\pi }}\frac{\textrm{SNR}_{D_0}}{\textrm{SNR}_D(m,n)}. \end{aligned}$$

For dark-field values *D≪ 1* the blur from the filtering dominates the achieved image resolution, and follows the relation $\sigma _{\textrm{filter}} \sim 1/D$.

**Fig. 3:**
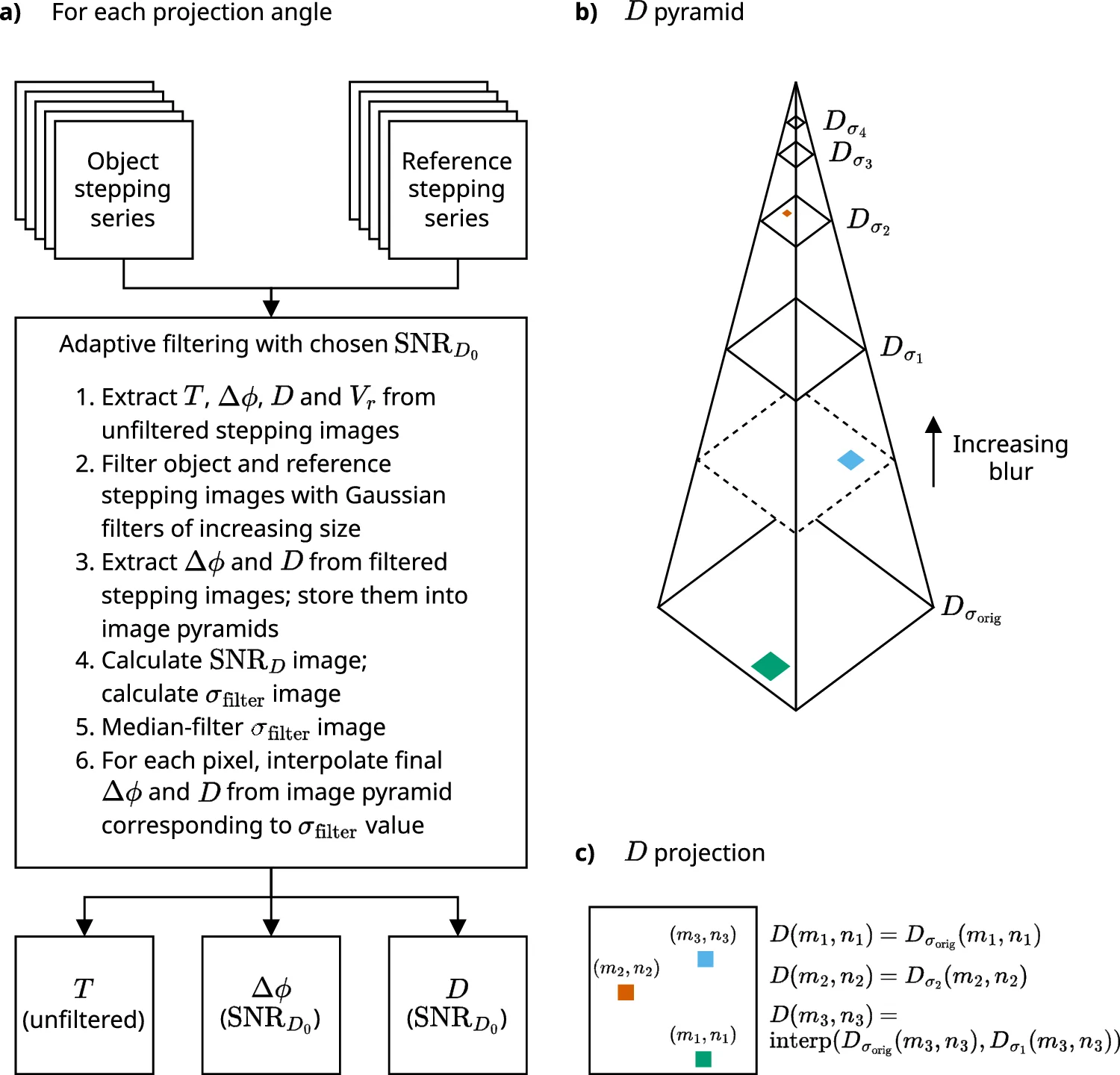
Adaptive filtering workflow. **a** Steps to create the filtered $\Delta \phi$ and *D* projections (corresponding to a chosen $\textrm{SNR}_{D_0}$ value) from a set of object and reference stepping images. The *T* image is extracted directly from unfiltered stepping images. The filtering is done individually for each tomographic angle. **b** Visualization of a Gaussian image pyramid for the dark-field signal. The first pyramid level corresponds to the unfiltered *D* projection, while each successive level corresponds to a filtered/blurred version of it, with increasing filter size (*σ*). **c** Each pixel of the final *D* projection is chosen from the image pyramid depending on the $\sigma _\textrm{filter}$ value for that pixel. For example, $\sigma _\textrm{filter}(m_2,n_2)$ now corresponds to the third level of the pyramid (i.e., $\sigma _2$), and thus $D(m_2,n_2)$ is taken from that pyramid level. Usually, $\sigma _\textrm{filter}(m,n)$ would not exactly correspond to the *σ* of a certain pyramid level, and thus the *D* value is interpolated from two nearby levels, as is the case with $D(m_3,n_3)$ in this example.

In order to solve for local $\sigma _{\textrm{filter}}$, we have to calculate the $\textrm{SNR}_D$, which itself depends on *D* (see equation (6)). For a noisy part of the image this calculation can be very imprecise, resulting in noise in both the $\textrm{SNR}_D$ and $\sigma _{\textrm{filter}}$ images. This would be undesired for the filtering, as the noise would propagate to the filtered projections. For this reason, we will work with a median-filtered version of the $\sigma _{\textrm{filter}}$ image (filter size 9-by-9 pixels). We will then filter the original stepping images (both object and reference) at each pixel with a Gaussian kernel corresponding to the desired value of $\sigma _{\textrm{filter}}(m,n)$. For efficiency reasons, however, we first filter both stepping image series with increasing values of the filter size, and store the resulting differential phase and dark-field images as Gaussian image pyramids (without sub-sampling)^44^. Then, at each pixel location (*m*, *n*), we linearly interpolate from these pyramids the $\Delta \phi (m,n)$ and *D*(*m*, *n*) values corresponding to $\sigma _{\textrm{filter}}(m,n)$. This process is repeated for each tomographic angle. As the transmission images do not suffer from the saturation of the visibility signal, *T* projections are calculated from unfiltered stepping images. The filtering workflow is visualized in Fig. 3.

### Experimental setup

The imaging setup used in this study is a three-grating Talbot-Lau interferometer system^1,45^. All gratings (Paul Scherrer Institute, Villigen PSI, Switzerland) are fabricated on a silicon substrate utilizing photolithography, etching and electroplating^46^. The G1 grating is a phase grating inducing a phase shift of *π* at 28 keV, with a period p$_1$=3.5 µm, line height h$_1$=36 µm, and duty cycle of 0.5. The G0 source grating and G2 analyzer grating have absorbing lines made of gold. The G0 period p$_0$=14 µm, line height h$_0$=50 µm, and duty cycle is 0.3. The G2 has a period p$_2$=2 µm, line height h$_2$=22 µm, and duty cycle of 0.5. The G0–G1 distance was set to 140 cm and G1–G2 distance to 20 cm, using the fifth fractional Talbot length. The X-ray tube (XRD Glass Tube, Malvern Panalytical B.V., Almelo, Netherlands) has a stationary tungsten anode, an effective focal spot size of 0.4 mm (v) *×* 0.8 mm (h) and a beryllium window with a thickness of 0.3 mm. The image detector (1207 CMOS, Varex Imaging Corporation, Salt Lake City, USA) is a flat panel detector with a gadolinium oxysulfide scintillator in front of a matrix of 1536 *×* 864 pixels with 74.8 µm pitch. The sample position is 5 cm in front of the G1 grating. The detector is placed 3 cm downstream of the G2 grating.

The sample, a mini-plum tomato (commercial product from a retail store), was imaged in a cylindrical plastic tube filled with water. The tube was attached from the top to a motorized rotating and translating stage. The X-ray tube voltage was set to 40 kV and the current to 40 mA. The phase stepping method^25^ was utilized to acquire samples of the phase stepping curves. The sample was rotated to 361 angles around 360$^\circ.$ At each angle, 10 raw images (1700 ms exposure time each) were acquired at equidistant phase stepping positions over one G2 grating period. Reference phase stepping sets were obtained at all angles, for which the sample was translated out of the field of view. The total imaging time was around 7 h.

Transmission, differential phase-contrast and dark-field images were retrieved from the phase stepping sets by applying a fast Fourier transform to the intensity modulations at each image pixel. The presented adaptive filtering process was applied to create filtered dark-field and phase-contrast projections at each tomographic angle. Filtered projections were created with different $\textrm{SNR}_{D_0}$ values. The image extraction and processing codes were implemented using MATLAB (version R2019b, The MathWorks Inc.).

A Feldkamp-Davis-Kress type algorithm (FDK_CUDA, ASTRA Toolbox^47,48^, Python) was applied to the transmission and dark-field projections for tomographic reconstruction. A one-dimensional integral (using a Fourier space filter^49^) was conducted on each row of the differential phase-contrast projections before reconstruction with the same FDK_CUDA algorithm.

## Results

**Fig. 4:**
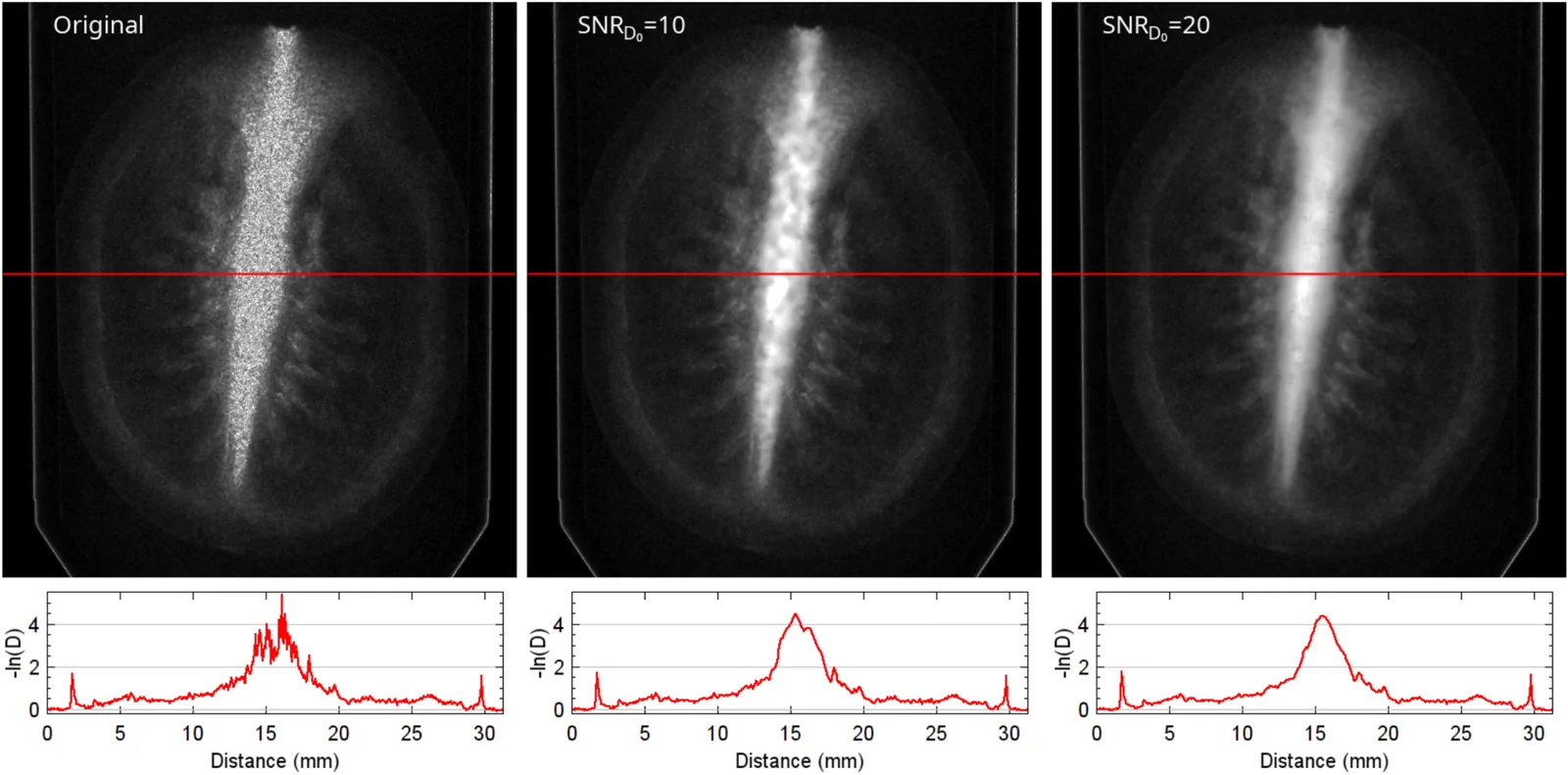
Original and filtered dark-field projections ($-\textrm{ln}(D)$) and corresponding line profiles of a mini-plum tomato in water. The general strength of the filter is adjusted by setting a minimum $\mathrm{SNR}$ value ($\textrm{SNR}_{D_0}$). The higher the $\textrm{SNR}_{D_0}$, the stronger the blurring. Locally, the strongest smoothing is obtained in the central region, which has the lowest $\mathrm{SNR}$ values, as shown in Fig. 5.

**Fig. 5:**
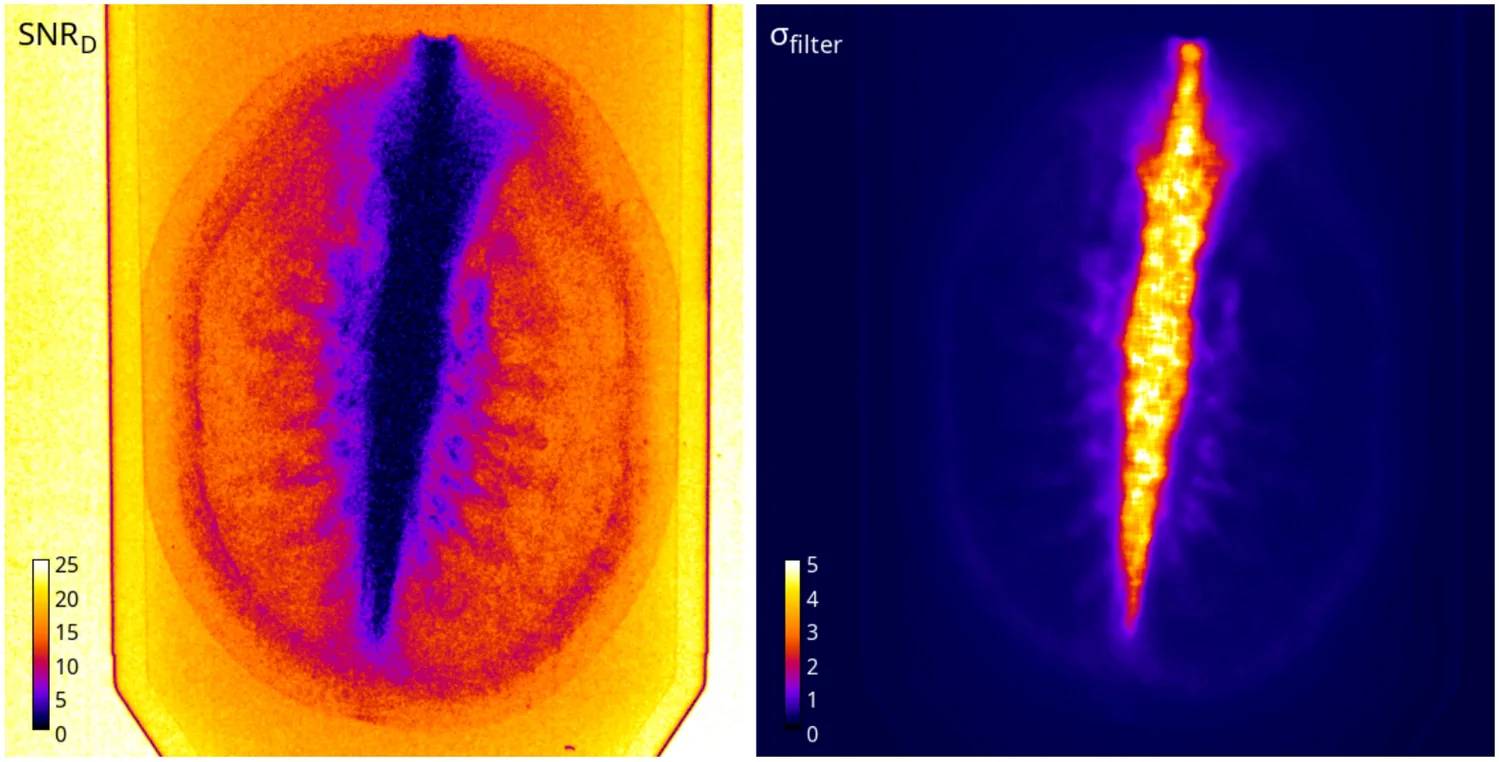
$\textrm{SNR}_D$ image, and $\sigma _{\textrm{filter}}$ image (with $\textrm{SNR}_{D_0}=10$), corresponding to the dark-field projection in Fig. 4. $\sigma _{\textrm{filter}}$ is calculated from $\textrm{SNR}_D$ using Eq. 12 and subsequently filtered with a moderately-sized (9-by-9 pixels) median filter to reduce noise.

Figure 4 shows an unfiltered (original) and two filtered dark-field projections of a mini-plum tomato sample. Different values of $\textrm{SNR}_{D_0}$ can be used in the proposed adaptive filtering method. The higher the $\textrm{SNR}_{D_0}$, the stronger the blurring/smoothing of the images, as a higher minimum SNR is required in the image. This can also be observed from the attached line profiles. The dark middle part of the tomato (columella) causes strong scattering of X-rays, and consequently the dark-field signal and $\mathrm{SNR}$ (see Fig. 5) in this region are very low. Thus, locally the strongest smoothing is obtained in the central region, while the sharpness in surrounding areas is maintained.

**Fig. 6:**
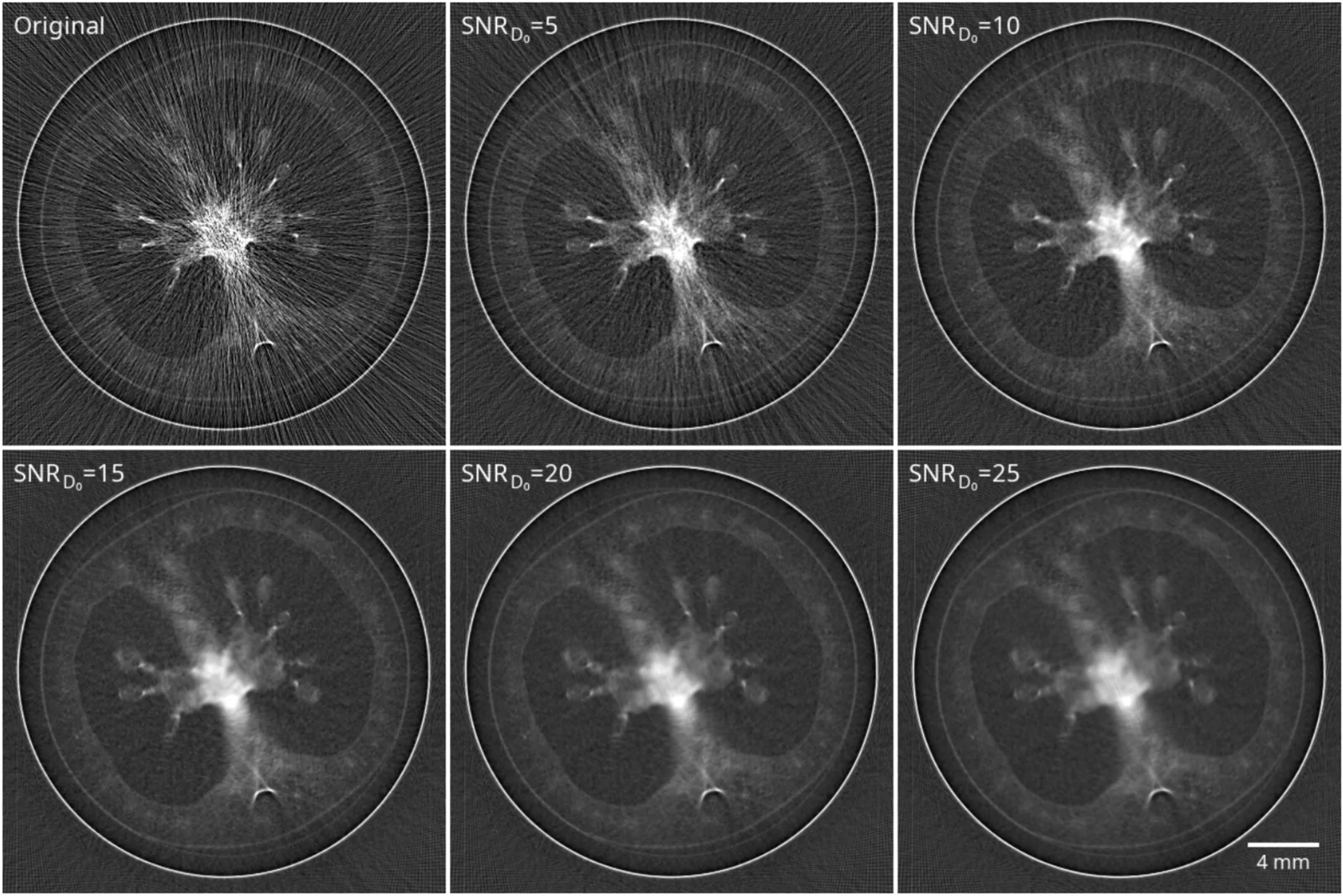
Axial slices from unfiltered (original) 3D dark-field data (*D*) and with different $\textrm{SNR}_{D_0}$ values used in the adaptive filtering. Gray value range: *[-0.01,0.04]*.

**Fig. 7:**
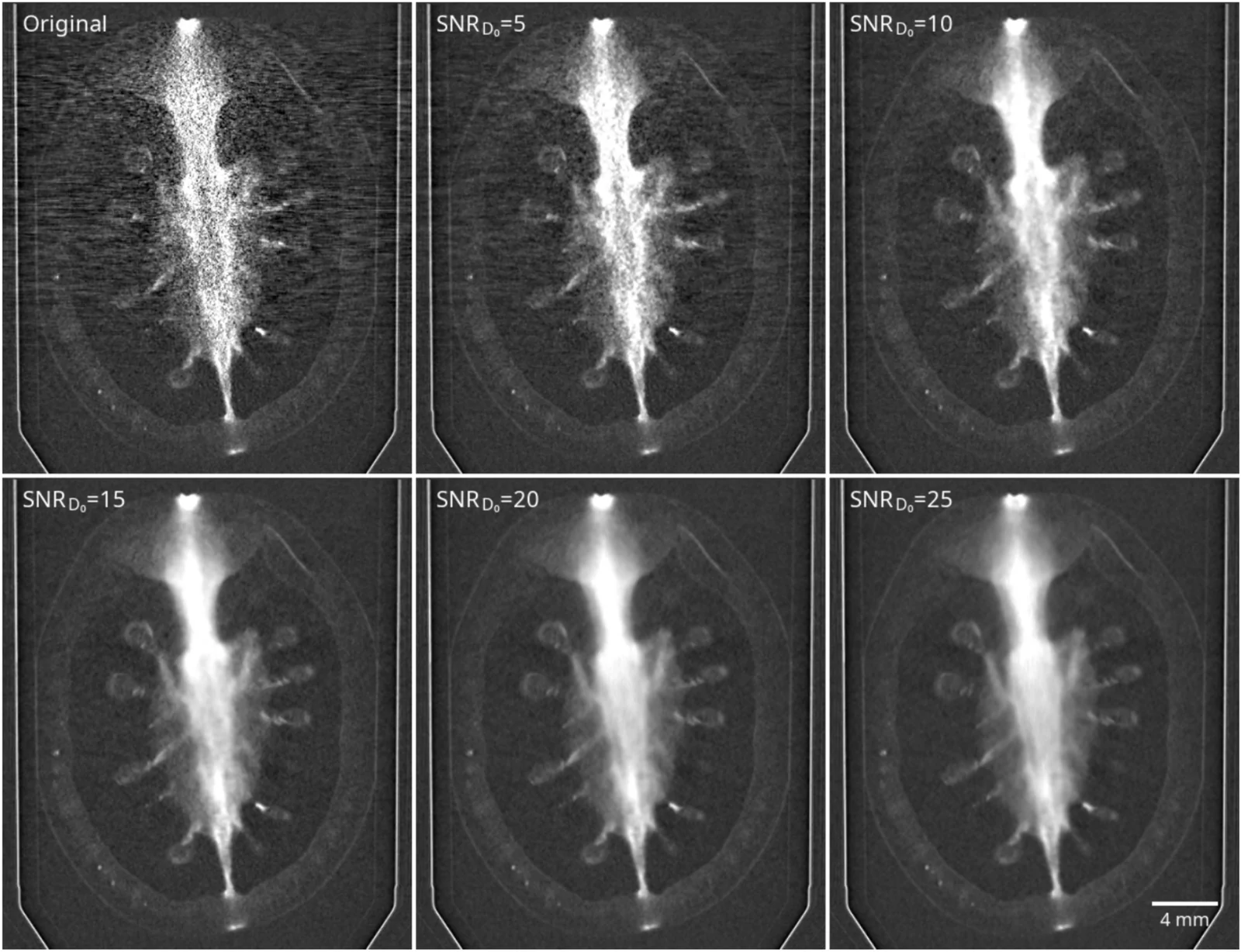
Lateral slices from unfiltered (original) 3D dark-field data (*D*) and with different $\textrm{SNR}_{D_0}$ values used in the adaptive filtering. Gray value range: *[-0.01,0.04]*.

Figures 6 and 7 show axial and lateral slices from the reconstructed dark-field volume obtained from unfiltered (original) and adaptively filtered projections with different $\textrm{SNR}_{D_0}$ values. The unfiltered axial slice suffers from heavy radial streak artifacts arising from the noise in the strongly scattering center of the tomato. In the lateral slice, heavy noise is obtained in the center region, and horizontal streaks go across the full width of the image. Notably, the strongest artifacts are present in areas where the columella is the thickest. Most of the features, such as the seeds, are overshadowed by the artifacts.

As observed with the projection images, higher values of $\textrm{SNR}_{D_0}$ result in generally stronger smoothing. Locally, the strongest smoothing is applied to the areas with lowest $\mathrm{SNR}$. In the reconstructed slices, this results in a reduction of the streak artifacts, more effectively with higher $\textrm{SNR}_{D_0}$ values. Thus, by varying $\textrm{SNR}_{D_0}$, a compromise between the amount of artifacts and strength of blurring can be found. Already with $\textrm{SNR}_{D_0}=10$ the reconstructed slices are much more usable than the unfiltered slices. Moreover, while the structures in the most strongly scattering areas (such as columella) start to alter because of the strong blurring at a high $\textrm{SNR}_{D_0}$, the structures of features in other areas (such as mesocarp) are largely unaffected.

**Fig. 8:**
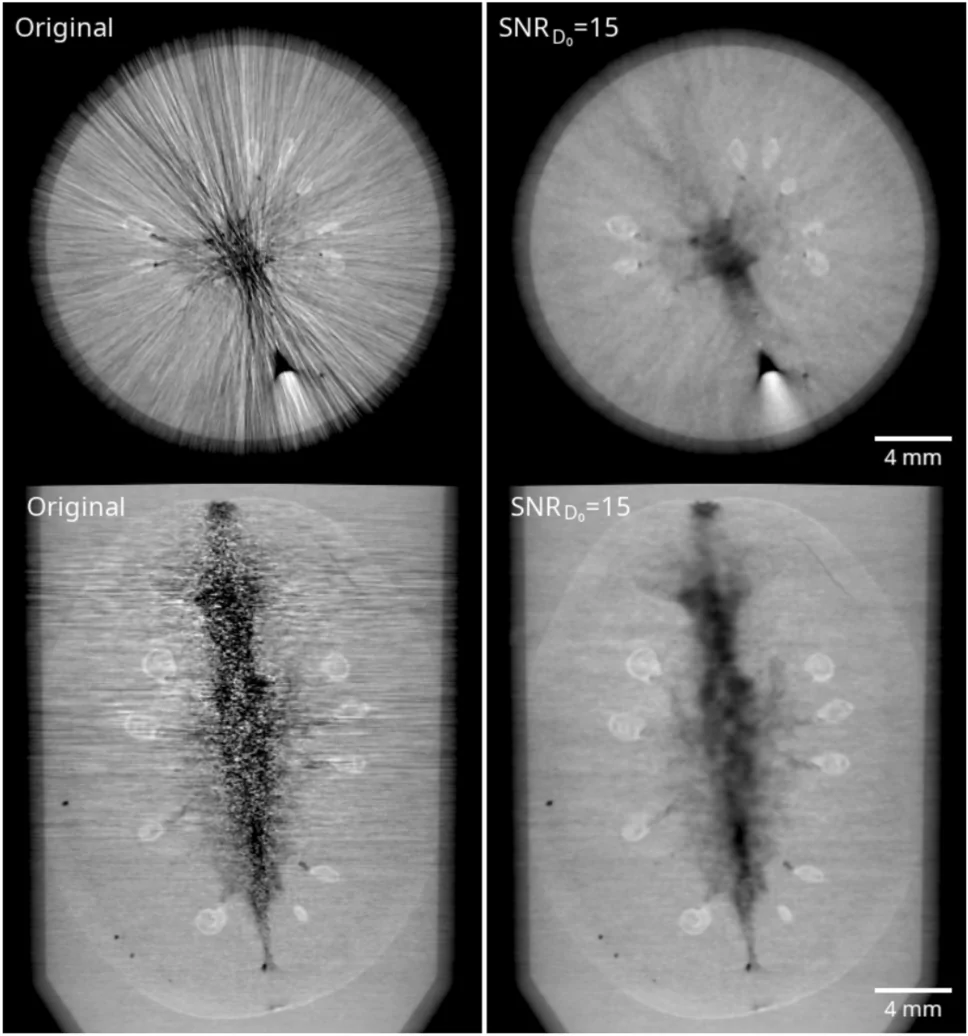
Axial (top) and lateral (bottom) slices from 3D phase-contrast data (integrated $\Delta \phi$) created from unfiltered projections (original) and filtered projections ($\textrm{SNR}_{D_0}=15$). While the contrast between different structures is generally lower than in the dark-field images, the streak artifacts are similar, and effectively reduced by the filtering. Gray value range: [1, 1.7].

Figure 8 shows corresponding phase-contrast slices obtained and processed simultaneously with the dark-field images. While the contrast between air and water/sample is much higher in the phase-contrast images, the contrast between the inner tomato features is higher in the dark-field images. The streak artifacts present in the dark-field slices (from unfiltered projections) are also present in the phase-contrast slices. They are effectively reduced by the adaptive filtering. A slight cupping artifact is additionally present in the phase-contrast slices, which is independent of the filtering.

## Conclusions

In this study, we investigated the effects of strongly scattering regions in a mini-plum tomato sample on X-ray dark-field and phase-contrast image data. In these regions, the visibilities of the local Talbot interference patterns are reduced to a point where the statistical noise becomes dominant. Thus, the sinusoidal shape of the fringes cannot be retrieved reliably, and the areas appear noisy in the dark-field and phase-contrast projections, which both rely on the visibility of the fringes. This translates into heavy radial and horizontal streaking artifacts in the axial and lateral reconstructed slices, respectively. These artifacts make the analysis and visualization of any adjacent weaker scattering features difficult or even impossible.

We presented an adaptive filtering method applied to the raw phase stepping images to reduce the scattering-induced artifacts in the processed data. Locally, the strength of the filtering is controlled by the local $\mathrm{SNR}$ of the dark-field image, and regions closer to saturation (SNR close to zero) are blurred more. The general strength of the filter can be adjusted by setting a minimum $\mathrm{SNR}$ value ($\textrm{SNR}_{D_0}$) for the image. The filtering method effectively reduces artifacts in both reconstructed dark-field and phase-contrast slices of the examined mini-plum tomato sample, making it possible to see features previously hidden behind the artifacts, such as the seeds.

The general weakness of image filtering methods is that strong blurring results in a significant reduction in spatial resolution. This blurring can thus hide or alter small features that otherwise would be detectable with the system resolution. In the case of the examined sample, however, the saturation-induced artifacts are so strong, that the reconstructed data is almost unusable without the filtering. Importantly, due to the adaptive nature of the filtering method, the strongest blurring is limited to the columella of the tomato, retaining features in the seeds and inner skin. The $\textrm{SNR}_{D_0}$ parameter can additionally be chosen to find a compromise between the strength of blurring and the amount of artifacts.

It is important to note that the presented filtering method has another tunable parameter in addition to $\textrm{SNR}_{D_0}$, namely the size of the median filter applied to the $\sigma _{\textrm{filter}}$ image corresponding to each projection. As we use the noisy *D* image in the calculation of $\textrm{SNR}_D$, also the $\sigma _\textrm{filter}$ image is noisy. Part of this noise will still remain in the adaptively filtered *D* (and $\Delta \phi$) image, unless we first smooth the $\sigma _\textrm{filter}$ image. The type and size of this smoothing filter affects the result of the adaptive filtering of the *D* and $\Delta \phi$ projections. The median filter utilized in this work was found to be a good compromise. In general, if this filter size is too small, noise will propagate to the adaptively filtered projections. If it is too large, the $\sigma _{\textrm{filter}}$ image will be distorted. As a result, parts of the projections would either be blurred too much or too little, as the smallest and largest $\sigma _{\textrm{filter}}$ values are filtered out.

Our image processing based method offers an important alternative in the case where other methods (for example, adjusting the autocorrelation length) are inconvenient or ineffective. Moreover, while a plethora of methods exist for avoiding the similar photon starvation artifacts, the approach we presented here is tailored towards grating-based dark-field and phase-contrast imaging, and the saturation of the visibility signal. In future research, the presented method should be further validated with well-quantified samples producing different levels of saturation of the dark-field signal. These could be, for example, microspheres of different sizes, or different thicknesses of a single scattering material.

## Data Availability

The datasets, including raw and processed images, generated during this study are available in the Fairdata IDA repository (CSC - IT Center for Science Ltd), https://doi.org/10.23729/fd-4ee62305-c5f3-3f0f-bd1f-1326ded97b69.

## Code availability

Code for generating the data supporting this work is available on GitLab^50^. The filtering function and the main program are in the files regularize_with_resolution_pyramid.m and extract_filtered_tomoseries.m, respectively. Compatible image reconstruction codes are also available on GitLab^51^. Please refer to the README.md files in the repositories for further instructions.
